# The bird-like complexity of hadrosauroid beaks

**DOI:** 10.1098/rsos.260392

**Published:** 2026-11-11

**Authors:** Marco Muscioni, Filippo Maria Rotatori, Alfio Alessandro Chiarenza, Diego Dreossi, Federico Fanti

**Affiliations:** ^1^ BiGeA, Alma Mater Studiorum University of Bologna, Bologna, Emilia-Romagna, Italy; ^2^ Collezione di Geologia ‘Museo Giovanni Capellini’, Bologna, Emilia-Romagna, Italy; ^3^ Institute of Geological Sciences, Freie Universität Berlin, Berlin, Germany; ^4^ GeoBioTec, Department of Earth Sciences, NOVA School of Science and Technology, Universidade Nova de Lisboa, Caparica, Portugal; ^5^ Museu da Lourinhã, Lourinhã, Portugal; ^6^ Department of Earth Sciences, University College London, London, UK; ^7^ Elettra Sincrotrone Trieste SCpA, Trieste, Friuli-Venezia Giulia, Italy

**Keywords:** *Tethyshadros*, hadrosauroid, beak, neurovascular system, rhamphotheca, thermoregulation, Cretaceous, microanatomy, Dinosauria, comparative anatomy

## Abstract

Duck-billed dinosaurs evolved complex cornified oral structures whose soft-tissue biology remains poorly understood. Here we investigate the rostral anatomy of the hadrosauroid *Tethyshadros insularis* using gross morphology and micro-computed tomography (micro-CT) data, focusing on the premaxillae and predentary. Structures previously interpreted as anomalous ‘spiked’ beak ornamentation are reinterpreted as osteological correlates of an extensive neurovascular network. Circumflex vessels, palatine and narial circulation and a median premaxillary sinus supplied a hypertrophied keratinous rhamphotheca that probably exceeded the extent of the underlying bone in both size and morphological complexity. Comparative evidence indicates that marginal denticulations reflect vascular excavation and/or ossified dermal papillary structures, implying vascular and somato-sensory functions analogous to avian beaks. Mesowear marks and functional correlates support a major role in cropping abrasive, grit-laden vegetation, while the dense vascularization suggests an additional thermoregulatory function via beak–nares heat exchange. These findings re-evaluate the hadrosauroid beak as a multifunctional sensory, mechanical and thermal organ, representing a key innovation that may have underpinned the ecological expansion and gigantism of later diverging hadrosaurids.

## 1. Introduction

Bird beaks are among the most complex and versatile oral structures in tetrapods. They consist of a cornified structure at the edentulous terminal portion of the rostrum, where a bony core supports a keratinous sheath called rhamphotheca; the rhamphotheca, which is divided into the rhinotheca (the upper portion, usually covering the premaxillae) and the gnathotheca (the lower portion, usually covering the dentary). Across more than 10 000 species, beaks show extreme variation in shape reflecting how birds feed, interact and move through their environments [1,2]. These structures also play key roles in preening, manipulation, thermoregulation, vocal production and even locomotion [2–5]. A flexible genetic substrate and developmental pathways allow a highly versatile, semi-independent regulation of beak characters, while still maintaining shared growth patterns that date back to non-avian theropod dinosaurs [6–9].

Beaks were not restricted to theropods, and multiple lineages of ornithischian dinosaurs evolved specialized beaks or beak-like structures [9–11] often in tandem with toothed jaws. Duck-billed hadrosauroid dinosaurs represent a clear example of this condition, with anterior edentulous beaks and posterior specialized tooth batteries for efficient mastication. However, the physiological role of the hadrosauroid beak, its *in vivo* appearance and its palaeoecological relevance have been somewhat overlooked [12]. The hadrosauroid *Tethyshadros insularis* offers an ideal opportunity to investigate this topic: in addition to being one of the best-preserved iguanodontian dinosaurs in Europe, this early Campanian hadrosauroid shows a seemingly unusual morphology of the premaxillary tomial edge [13,14], bearing forward-facing rod-like denticles. These structures were originally regarded as a distinctive feature of this taxon and evidence of a unique comb-like toothed beak [13], an interpretation also reflected in popular artistic reconstructions. More recently, *T. insularis*, *Telmatosaurus* and *Kryptohadros* were grouped in the clade Telmatosauridae, an endemic southeastern European lineage of hadrosauroids, and the presence of three denticles on the anteroventral edge of the premaxilla was included among the diagnostic characters of the clade [15].

Here we provide a detailed description of the microanatomy of the premaxillae and predentary bones of the *T. insularis* holotype, integrating macromorphology and micro-CT to reconstruct the neurovascular network of the rostral region. Through comparisons with other extinct and extant taxa, we reinterpret the denticulations as osteological correlates of specific soft tissues. Our results indicate a complex multifunctional role of the rostral beak/naris complex with immediate relevance to rhamphothecal growth and morphology, sensory abilities and the thermophysiology of hadrosauroids in general.

### Institutional abbreviations

AMNH
: American Museum of Natural History, New York, USA
CM
: Carnegie Museum of Natural History, Pittsburgh, Pennsylvania, USA
CMN
: Canadian Museum of Nature, Ottawa, Ontario, Canada
JRF
: Judith River Foundation/Judith River Dinosaur Institute, Malta, Montana, USA
LACM
: Natural History Museum of Los Angeles County, Los Angeles, California, USA
MCSNT
: Museo Civico di Storia Naturale di Trieste, Trieste, Italy
MPC
: Mongolian Palaeontological Centre, Institute of Paleontology, Mongolian Academy of Sciences, Ulaanbaatar, Mongolia
MSC
: McWane Science Center, Birmingham, Alabama, USA
NHMUK
: Natural History Museum, London, UK
OUVC
: Ohio University Vertebrate Collection, Athens, Ohio, USA
PIN
: Palaeontology Institute Nauk, Moscow, Russia
RAM
: Raymond M. Alf Museum of Paleontology, Claremont, California, USA
ROM
: Royal Ontario Museum, Toronto, Ontario, Canada
SC
: Italian State Collection (Trieste, Italy)
SMF
: Naturmuseum Senckenberg, Frankfurt, Germany
TMM
: Texas Memorial Museum, Austin, Texas, USA
TMP
: Royal Tyrrell Museum of Palaeontology, Drumheller, Alberta, Canada
UALVP
: University of Alberta Laboratory of Vertebrate Paleontology, Edmonton, Alberta, Canada
YPM
: Yale Peabody Museum, New Haven, Connecticut, USA

## 2. Material and methods

### 2.1. Specimens

We analysed three individuals of *T. insularis*: SC 57021 (holotype), SC 57247 and SC 57026 [13,14] (figure 1). The holotype is virtually complete and represents a somatically immature individual (figure 1*a*); SC 57247 is a larger, articulated specimen lacking limbs (figure 1*c*); SC 57026 is a large individual (up to 130% the size of the holotype) represented by pervasively fragmented but articulated skull and some limb bone fragments [13,14] (figure 1*e*). As they are preserved in laminated limestone, all specimens were prepared by chemical dissolution at ZOIC S.R.L. (Trieste) with 5% formic acid, a methodology that significantly influenced the quality of the CT data presented here. Both premaxillae, the distal portions of the dentaries and the predentary of the holotype were entirely extracted from the matrix through chemical dissolution. All specimens are housed at the Museo Civico di Storia Naturale di Trieste (MCSNT, herein listed with the State Collection code SC).

**Figure 1.**
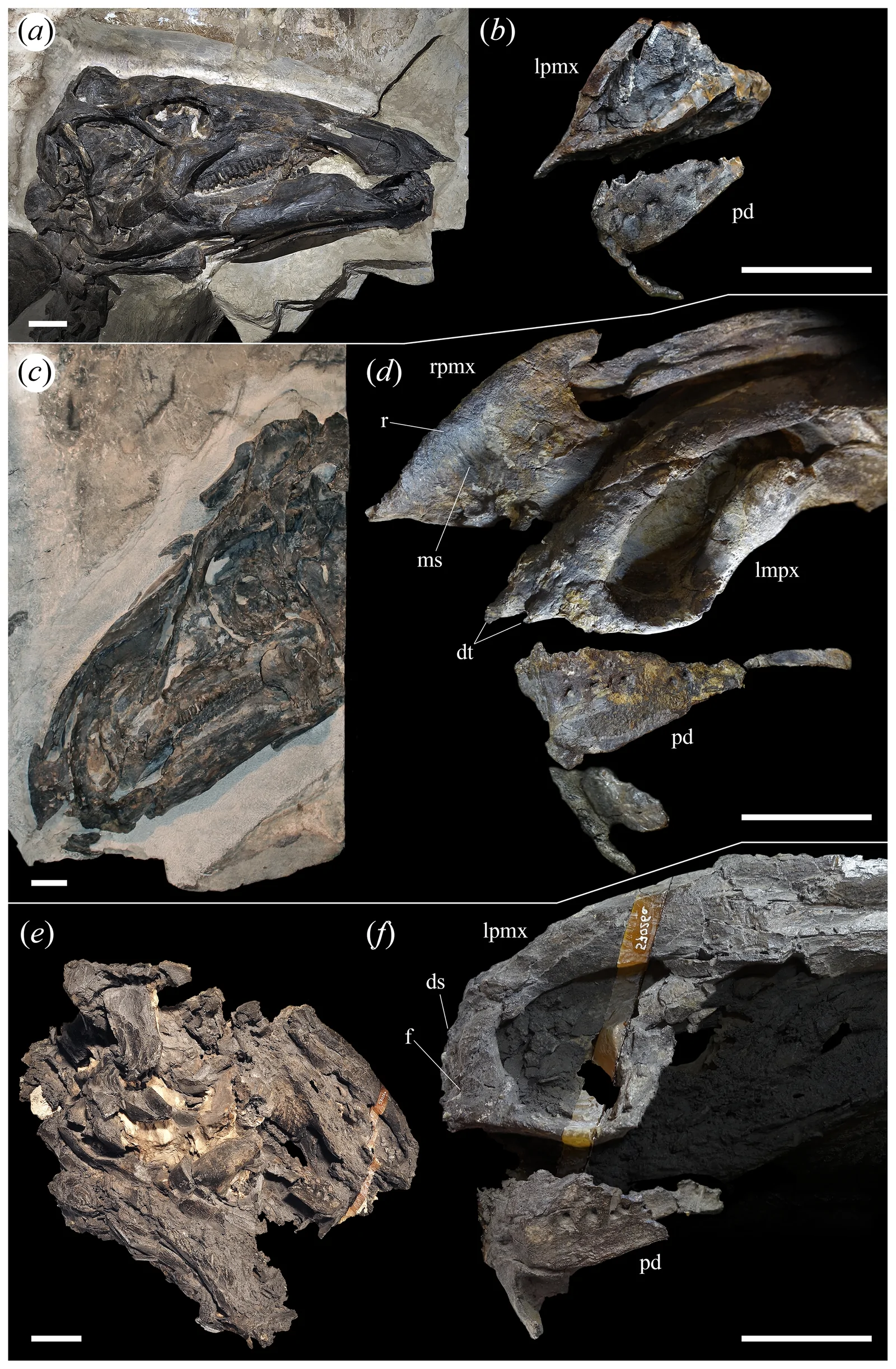
Comparison of the three *Tethyshadros* specimens. (*a*) Skull of SC 57021; (*b*) detail of the premaxillae and predentary in left lateral view; (*c*) skull of SC 57247; (*d*) detail of the premaxillae and predentary in left lateral view; (*e*) skull of SC 57026 in right lateral view; and (*f*) detail of the premaxilla and predentary in right lateral view, mirrored to be directly comparable to the other specimens. Abbreviations: dt, denticles; f, foramen; lpmx, left premaxilla; ms, medial sinus; pd, predentary; r, rugose surface; rpmx, right premaxilla. Scale bars = 50 mm.

### 2.2. Tomography

Tomographic analysis focused on the rostralmost part of the premaxillae and predentary of SC 57021. Four acquisitions were carried out at the X-ray microtomography laboratory TomoLab Elettra (FAITH; Basovizza, Trieste) with the following parameters: X-ray source (Hamamatsu L12161-07) settings: HV = 145 kVp; *I* = 450 µA, focal spot = 50 µm. Filters: 2 mm copper plus 2.25 mm brass. Detector: Dalsa Shad-O-Box 6k HS, matrix 2940 × 2304, pixel size 49.5 µm and active area of 145 × 114 mm^2^. Geometry: source distance = 380 mm, detector distance = 627 mm, equivalent pixel 30 µm. Local Area CT acquisition: 3600 projections with exposure time of 4 s. Reconstruction software: NRecon v.1.6.9.18, Bruker. Segmentation was done using Avizo3D. These CT scans are available on the online repository MorphoSource, Media IDs 000821984 (premaxillae) and 000822290 (predentary) alongside the derived 3D mesh models of the bones and neurovasculature.

### 2.3. Comparative specimens

In addition to data drawn from the literature, the specimens in table 1 were examined first-hand.

**Table 1.** Comparative specimens examined first-hand.

taxon	specimen code	element of interest
*Daspletosaurus torosus*	TMP 2001.036.0001	skull
*Deinocheirus mirificus*	MPC-D 100/127	skull
*Ornithomimus edmontonicus*	TMP 1995.110.0001	skull, rhamphotheca (cast)
*Struthiomimus* sp.	TMP 1990.026.0001	skull
*Borealopelta markmitchelli*	TMP 2011.033.0001	skull, rhamphotheca
*Edmontonia rugosidens*	TMP 1998.098.0001	skull
Ankylosauridae indet.	TMP 2025.012.0432	skull
*Tsagantegia longicranialis*	MPC-D 700/17	skull
*Centrosaurus apertus*	TMP 1994.182.0001	skull
*Triceratops horridus*	TMP 2014.022.0022	skull
Styracosterna indet.	NHMUK PV R102X	predentary
*Dryosaurus elderae*	CM 11340	skull
*Camptosaurus dispar*	YPM 1880	premaxillae
*Tenontosaurus tilletti*	YPM 5456	skull
*Gobihadros mongoliensis*	MPC-D 100/754	skull
	MPC-D 100/763	skull
*Saurolophus angustirostris*	MPC-D 100/706	skull
	MPC-D 100/764	skull (cast)
*Prosaurolophus maximus*	TMP 1998.050.0001	skull
*Brachylophosaurus canadensis*	TMP 1990.104.0001	skull
*Edmontosaurus regalis*	ROM 801–HCF	skull (cast)
Saurolophinae indet.	TMP 2013.043.0001	skull, rhamphotheca
*Hypacrosaurus stebingeri*	TMP 1989.079.0052	skull
*Hypacrosaurus altispinus*	TMP 2013.025.0001	skull, rhamphotheca
cf. *Hypacrosaurus* sp.	UALVP 57103.01	skull
*Lambeosaurus magnicristatus*	UALVP 60000.00	skull
Hadrosauridae indet.	TMP 1991.036.0652	predentary
Hadrosauridae indet.	TMP 1981.027.0050	predentary

Osteological specimens of extant taxa were either housed in the Comparative Anatomy Collection of the University of Bologna or the Ornithology Collection (Museum of Zoology) of the University of Alberta or were personally prepared by the authors for this study. Osteological preparation of fresh specimens was performed by water maceration, followed by degreasing with diluted acetone and whitening with hydrogen peroxide. To inspect the dermal layer, rhamphothecae were removed from the skulls and partially bleached with 15% hydrogen peroxide. Specimens of the following taxa were used as our comparative sample: *Caretta caretta* (skull), *Pelodiscus sinensis* (skull and rhamphotheca), *Crocodylus siamensis* (skull), *Alligator mississippiensis* (skull), *Tomistoma schlegelii* (skull), *Rhea americana* (skull), *Dromaius novaehollandiae* (skull), *Anser cygnoides* (skull and rhamphotheca), *Chen caerulescens* (skull and rhamphotheca), *Gallus gallus* (skull and rhamphotheca), *Phasianus colchicus* (skull and rhamphotheca), *Numida meleagris* (skull and rhamphotheca), *Phalacrocorax olivaceus* (skull), *Diomedea* sp. (skull), *Ardea herodias* (skull), *Coloeus monedula* (skull), *Corvus corax* (skull), *Equus ferus* (manual ungual and keratin sheath) and *Bos taurus* (pedal unguals and keratin sheaths). The papillae and rhamphotheca of *Ara macao* were segmented from publicly available CT scans of specimen OUVC 10633. WitmerLab at Ohio University provided access to these data originally appearing on The Visible Interactive Parrot website, the collection of which was funded by NSF. The files were downloaded from www.MorphoSource.org, Duke University. Skull media ID: 000014113, mandible media ID: 000014108.

## 3 Results

### 3.1. Rostral osteology of *T. insularis*

#### 3.1.1. Premaxilla

In the holotype specimen SC 57021, both premaxillae are broken vertically at roughly the level of the proximal edge of the naris, where large portions of the nasal and caudolateral processes are missing (figures 1 and 2). Both premaxillae preserve the anterior portion of the nasal processes, which forms the rostral margin of the external naris. The good preservation of this feature in specimens SC 57247 and SC 57026 allows the recognition of a more ventrally curved dorsal outline of the nasal processes than inferred from the holotype [13] (figure 1). The right posterior portion of both processes is articulated with the right maxilla and nasals. Premaxillae are more mediolaterally compressed in the larger specimens SC 57247 (15–20% larger than the holotype) [14] and SC 57026 (20–30% larger than the holotype) (figure 1). Left and right elements are unfused in each of the three specimens, accounting for their slightly disarticulated state (figure 1). The right premaxilla in SC 57021 is also heavily mediolaterally compressed, to the point that its lateral surfaces appear almost subvertical; the left element, however, is minimally deformed, showing a significantly wider rostral margin in dorsal view (figure 2). The ventrolateral margin of the naris in the holotype, especially that of the left premaxilla, exhibits a broken squared vertical surface, suggesting a significant and previously underestimated laterodistal width. The anteriormost end of the premaxillae consists of a subtriangular structure whose dorsal surface hosts large foramina organized in at least one diagonal row (figure 2). The tomial edge of the premaxillae is equipped with anteriorly oriented ‘bony projections’ or oral denticles, three per side, which are progressively more developed rostrally. They are relatively thin and elongated in SC 57021, while they are thicker, blunt and more ventrally oriented in the larger SC 57247 (figure 1). Both premaxillae in the largest specimen (SC 57026) lack the anteriormost portion of the oral margin where the bony projections would have developed. Denticles on the premaxillae are variably identifiable in closely related taxa, including non-hadrosaurid hadrosauroids and hadrosaurids [16–23]. On the compressed right premaxilla of the holotype, these structures resemble conical rods projecting radially from the oral margin. However, in the less deformed left premaxilla, the projections are instead separated by deep indentations along a clearly identifiable tomial edge (figure 2). The lateral margins of the projections themselves are irregular and are marked by numerous semicircular depressions (figure 2*f*). The medial surface of the premaxilla is exposed in both SC 57021 (left side) and SC 57247 (right side), showing a concavity surrounded by a rugose outline, indicating where the paired element would have articulated through fibrous ligaments (figures 1*b* and 2*a*). The deepest part of the concavity is located at the anterior portion of the symphyseal surface, immediately behind the third and longest denticle, coincident with two deep pits or large horizontally oriented foramina. The palatal surface of the premaxillae bears wrinkled longitudinal sulci (figure 2*e*). A row of foramina smaller than the dorsal ones is visible near the premaxillary edge on its anterolateral margin. Caudal to these foramina, a secondary ridge parallel to the premaxillary margin is recognizable, probably homologous with the secondary caudal denticles observable in other taxa (e.g. *Prosaurolophus*, TMP 1998.050.0001; *Hypacrosaurus*, UALVP 57103.01; *Eotrachodon*, MSC 7949 [20]; *Magnapaulia*, LACM 17715 [16]; *Gryposaurus* TMM 46033-1 [24]). The remaining palatal surface is relatively smooth, with a deep, progressively sharper groove culminating in a subhorizontal rostrally oriented foramen entering through the palate near the medial margin of the premaxilla.

**Figure 2.**
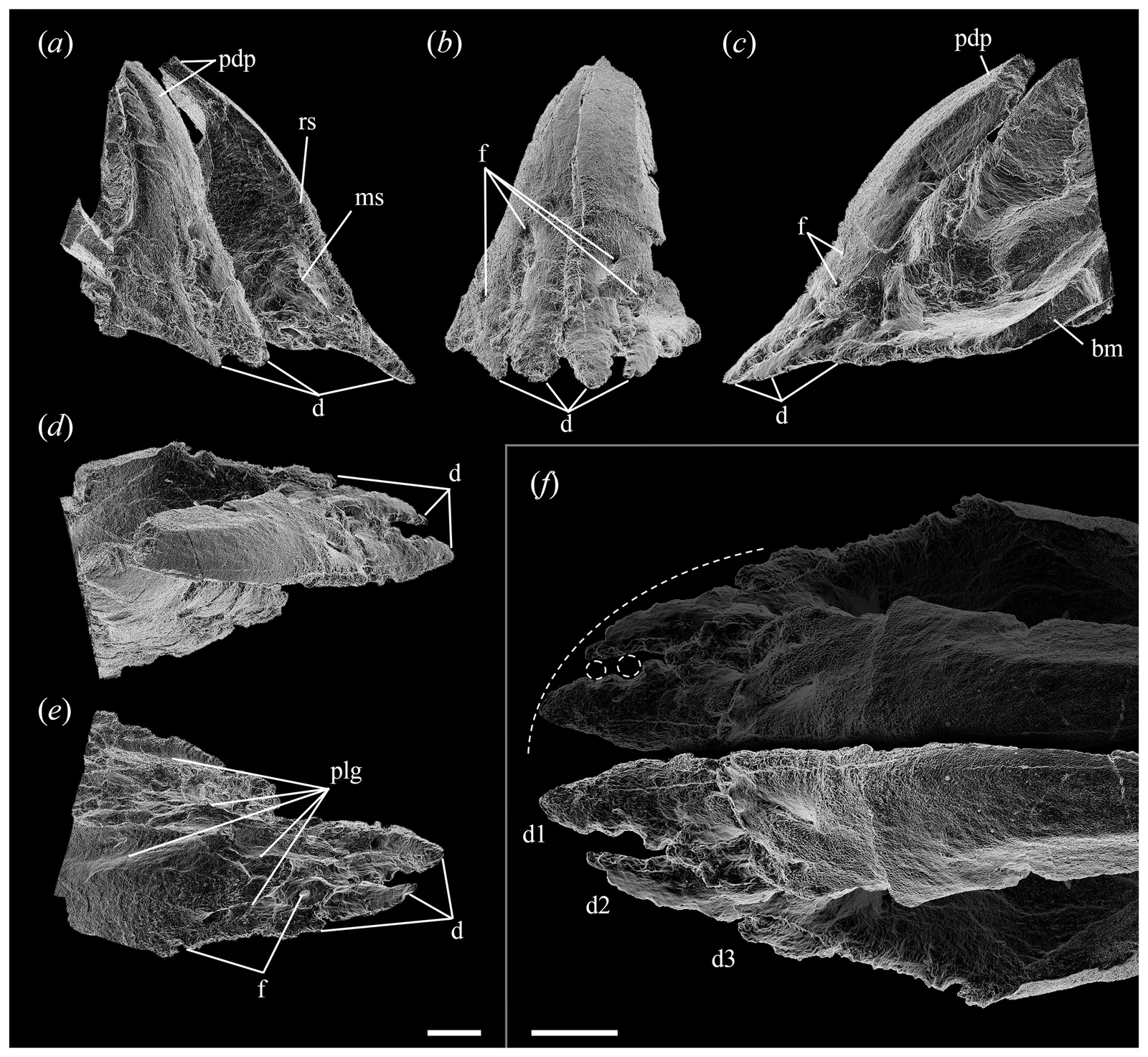
3D render of the paired SC 57021 premaxillae in (*a*) right lateral view, (*b*) anterior view, (*c*) left lateral view, (*d*) dorsal view and (*e*) ventral view. (*f*) Detail of the comparatively less deformed left premaxilla in dorsal view, paired with a mirrored version (darker tone) to replicate the original width of the rostral and narial region. Notice that premaxillary denticles describe a continuous tomial edge (dotted line) and preserve subcircular indentations along their margins (dotted circles). Abbreviations: bm, broken margin; d, denticle, denticles; f, foramina; ms, medial sinus; pdp, posterodorsal processes; plg, palatal grooves; rs, rugose surface. Scale bars = 10 mm.

#### 3.1.2. Predentary

The predentary of SC 57021 is three-dimensionally exposed and virtually complete, except for the posterior margin of the left ramus (figures 1 and 3). Mediolateral compression affected mainly its anteriormost portion, resulting in a narrow U-shaped profile in occlusal view (figure 3). The lateral surfaces are subvertical and perforated by at least eight large foramina per side. Lingually, these foramina are connected by a longitudinal groove (figure 3). The anteriormost foramen on both sides is slightly larger than the others, and two vertical labial grooves of comparable width connect the foramina with the dorsolateral surfaces of the large bilobate ventral process, leading ventrally below the element. The ventral contact surfaces for the dentaries are mediolaterally wide and distinctly flat, forming an almost 90° angle with the lateral surface. The preservation of the anteriormost right dentary up to the first tooth shows that no diastema was present between the caudal extremity of the predentary and the rostral limit of the tooth row. The posterior portion of the caudolateral processes bears a thin mediolateral expansion (figure 3). The tomial edge bears a bifurcated medial denticle and at least six progressively smaller and less defined triangular denticles on each side. The first denticle on each side is the largest and most prominent of the series, with an almost rhomboidal shape owing to the deep basal grooves separating it from the medial and third denticles (as in taxa such as *Brighstoneus simmondsi* [25] and *Equijubus normani* [26]). The predentaries of SC 57247 and SC 57026 are larger but morphologically consistent with the holotype (figure 1*d*,*f*). That of SC 57247 is heavily damaged: the left and right sides are separated along a vertical medial fracture, the bilobate process is separated and incomplete, and the overall smooth morphology of the tomial edge results from restoration for display purposes. The predentary of SC 57026 is undamaged and morphologically identical to that of SC 57021.

**Figure 3.**
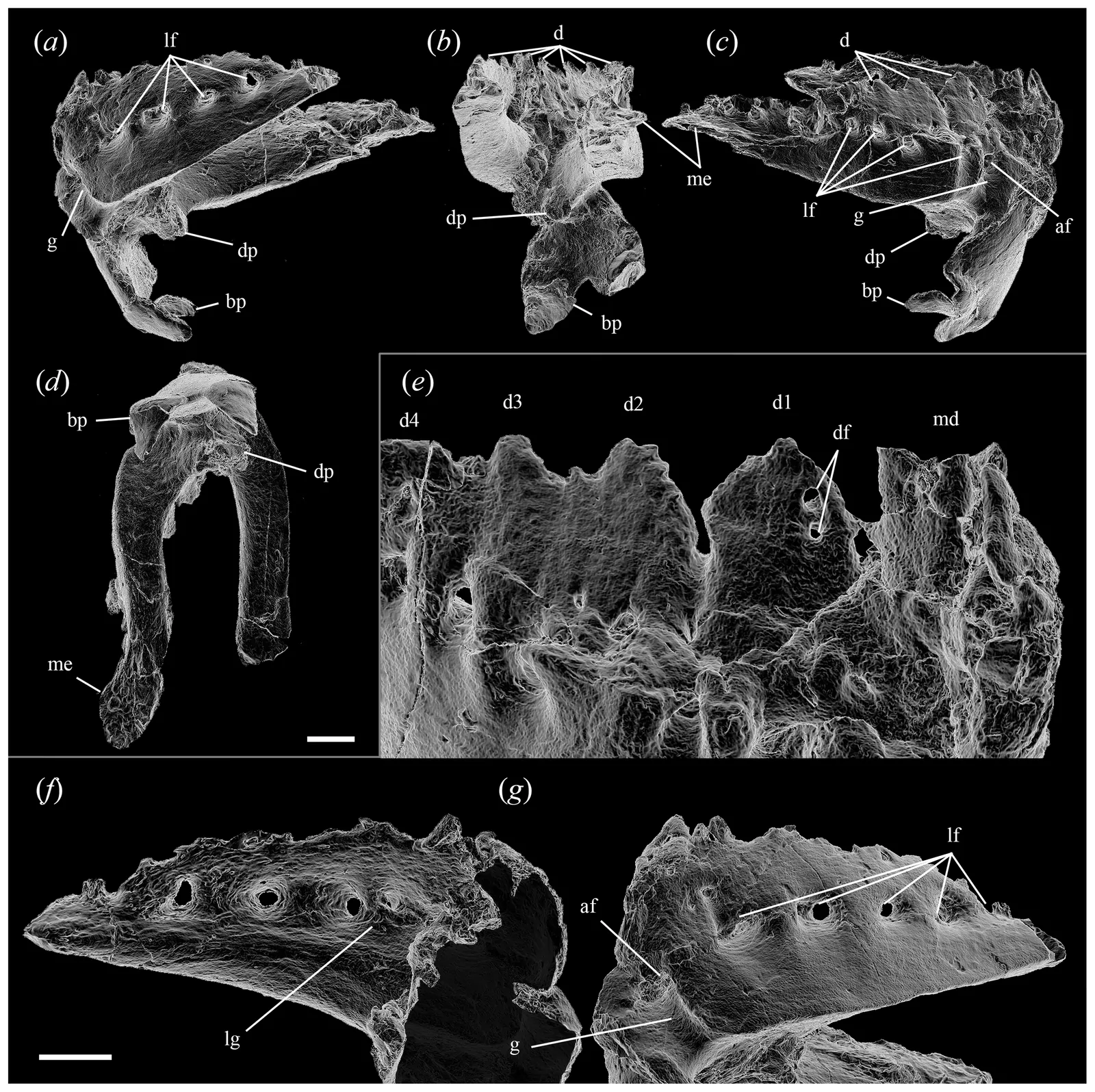
3D render of the SC 57021 predentary in (*a*) left lateral view, (*b*) posterior view, (*c*) right lateral view and (*d*) ventral view. (*e*) Detail of the triangular denticles on the right side. (*f*) Detail of the medial surface of the left ramus. (*g*) Detail of the lateral surface of the left ramus. Abbreviations: af, anterior foramen; bp, bilobate ventral process; d, denticles; dp, median dorsal process; g, anterior groove; lf, lateral foramina; lg, lingual groove; me, mediolateral expansion. Scale bars = 10 mm.

### 3.2. Comparative osteology

#### 3.2.1. Rostral anatomy in Dinosauria

Toothed saurischians, including both sauropodomorphs and theropods, often display extensive neurovascular correlates such as foramina and associated vascular grooves extending towards the oral margin on premaxillae and dentaries [27] (figure 4*a*). Sauropods and toothed theropods are traditionally not considered examples of beaked animals; however, the possibility of a premaxillary keratin sheath was notably proposed for dromaeosaurids [28] and a growing body of research is highlighting the likelihood of rhamphotheca-like cornified structures coexisting with teeth [11,29–31]. The presence of a rhamphotheca is widely accepted and sometimes even preserved in edentulous non-avian theropods such as ornithomimosaurs (e.g. *Ornithomimus edmontonicus*, TMP 1995.110.0001). *Deinocheirus* bears several aligned foramina with associated vascular grooves at the anterior extremities of the premaxillae and dentary, but the grooves do not incise the tomial edge deeply enough to produce indentations. Derived ornithomimids, such as *Ornithomimus*, *Struthiomimus* and *Gallimimus*, share the same pattern of aligned foramina and grooves concentrated on the anterior premaxillae and dentary (figure 4*b*). The large grooves directed towards the tomial edge create a fluting which could be interpreted in some specimens as incipient crenulations. This pattern is extremely similar to that of living birds such as ratites and large-bodied Galloanserae (figure 5; supplementary material, figure S2). Remarkably, a specimen of *Gallimimus* (MPC-D 100/1133) retains structures on the lateral margin of the rhinotheca that are reminiscent of the lamellae on the beaks of anseriforms [32]. Most non-avian theropods with beaks lack prominent indentations on the tomial edge [32], while others show small vascular indentations and/or true denticles (e.g. oviraptorids [33]). Among ornithischians, ceratopsids are unique in having the rhinotheca supported by the rostral bone, an unpaired element articulated anterior to the premaxillae; in Ceratopsidae, both the rostral and the predentary show almost no variation in neurovascular pattern, having an extremely high density of pervasive foramina and associated shallow anastomosing grooves on the whole external surface (figure 4*c,d*). The tomial edge in ceratopsids is rugose, owing to a multitude of foramina and associated grooves opening directly on the margin. Bone texture, neurovascular organization and occlusal morphologies in Ceratopsidae closely resemble those of extant chelonians. Ankylosaurs show a spectrum of conditions. *Tsagantegia* has numerous aligned foramina and associated grooves on the predentaries (figure 4*e*). Each groove extends to an indentation along the tomial edge, and the indentations are separated from each other by subtriangular denticles very similar to those present in Hadrosauriformes. On the other hand, *Edmontonia* bears a single large foramen on the dorsolateral surface of each premaxilla, surrounded by two to three smaller foramina (figure 4*f*). Each foramen is associated with a deep groove anastomosing with several others and extending to the tomial margin. The tomial margin is continuous except for a pair of anterior foramina creating a symmetrical indentation, presumably where most of the other blood vessels drained. Among beaked ornithopods, morphologies like the indentations on the tomial edge of premaxillae and predentaries of *Tethyshadros* are probably underreported. These thin structures may be widespread but are easily damaged or lost through both taphonomic processes and fossil preparation. In addition, they are often overlooked by researchers because variation in this anatomical feature has yet to be properly included in a phylogenetic analysis. Iguanodontians display higher variability than hadrosauroid taxa; for example, the predentary of certain styracosternans bears thin rod-like denticles and inconspicuous foramina (supplementary material, figure S1), while *B. simmondsi* has a highly perforated, hadrosauroid-like predentary with broad and well-developed denticles [25]. *Jinzhousaurus yangi* and *Bolong yixianensis* possess similarly perforated predentaries [34,35] although these are more distorted and therefore difficult to assess. Similarly, premaxillary denticles and arching vascular grooves are inconspicuous in many iguanodontians [36] (e.g. *Tenontosaurus*, supplementary material, figure S1), but a distinct vascular groove continuous with a large premaxillary foramen is visible in *Camptosaurus* (figure 6*a*). Non-hadrosaurid hadrosauroids are more homogeneous, with the premaxillary denticulation frequently present, albeit variable in size and proportions (e.g. *Eolambia* [17]; *Telmatosaurus* and *Kryptohadros* [15]). In taxa that lack predentary denticles (e.g. *Protohadros* [37]), the tomial margin is visibly scalloped. The same occurs in the perforated predentary (e.g. *Probactrosaurus* [38]), with the typical condition exemplified by the exquisitely preserved *Gobihadros* [21] (figure 6*b*). In *Gobihadros*, the premaxillary denticulations are separated by grooves which are clearly associated with foramina and, partly, to the vascular grooves inside the bony naris. Despite derived morphologies (e.g. dorsoventrally flattened premaxillae and broad, shallow predentaries) Hadrosauridae retain these characters, which are sometimes remarkably developed and easily recognizable (e.g. *Eotrachodon* [20]; *Hypacrosaurus*, *Lambeosaurus*, *Prosaurolophus*, *Brachylophosaurus*, *Saurolophus*, figure 6*c–h*). The main difference lies in the more ventrally placed denticles in saurolophines, which are often located under the labrum and resemble conical bumps (e.g. kritosaurines [24,39]; *Saurolophus*, figure 6*h*). In this regard, the condition observed in *Tethyshadros*, considering the variability among the three available specimens, does not appear to be exceptional or significantly different from other hadrosauroids. Moreover, the morphology and development of denticles are probably influenced by ontogeny and individual variation. Embryonic and perinatal hadrosaur individuals have strongly reduced or absent denticulations on both predentaries and premaxillae [40,41] (e.g. *Saurolophus angustirostris* MPC-D 100/764 [40], *Hypacrosaurus stebingeri* TMP 1989.079.0052, figure 6*c*), but these probably appear already in the earliest juveniles [42]. Immature/subadult specimens seemingly (but not always: e.g. subadult *Gobihadros mongoliensis* MPC-D 100/754, juvenile cf. *Hypacrosaurus* sp. UALVP 57103.01 figure 6*b*,*d*) have more defined premaxillary denticles, while in hyperossified adults the denticles sometimes thicken mediolaterally, the premaxillae increase their marginal thickness, and the entire denticulation may be reduced to low bumps or a wavy edge [20,43–45] (e.g. *S. angustirostris* MPC-D 100/706, figure 6*h*). In contrast, older individuals exhibit larger predentary denticles [46,47] (supplementary material, figure S1). These morphologies have already been documented in some Hadrosauridae with known ontogenetic series (e.g. *Edmontosaurus* [43]; *Kritosaurus* [45,46]) but are also directly visible in *Tethyshadros*, as the younger SC 57021 bears much more defined and relatively more prominent denticles than the older SC 57247 (figure 1).

**Figure 4.**
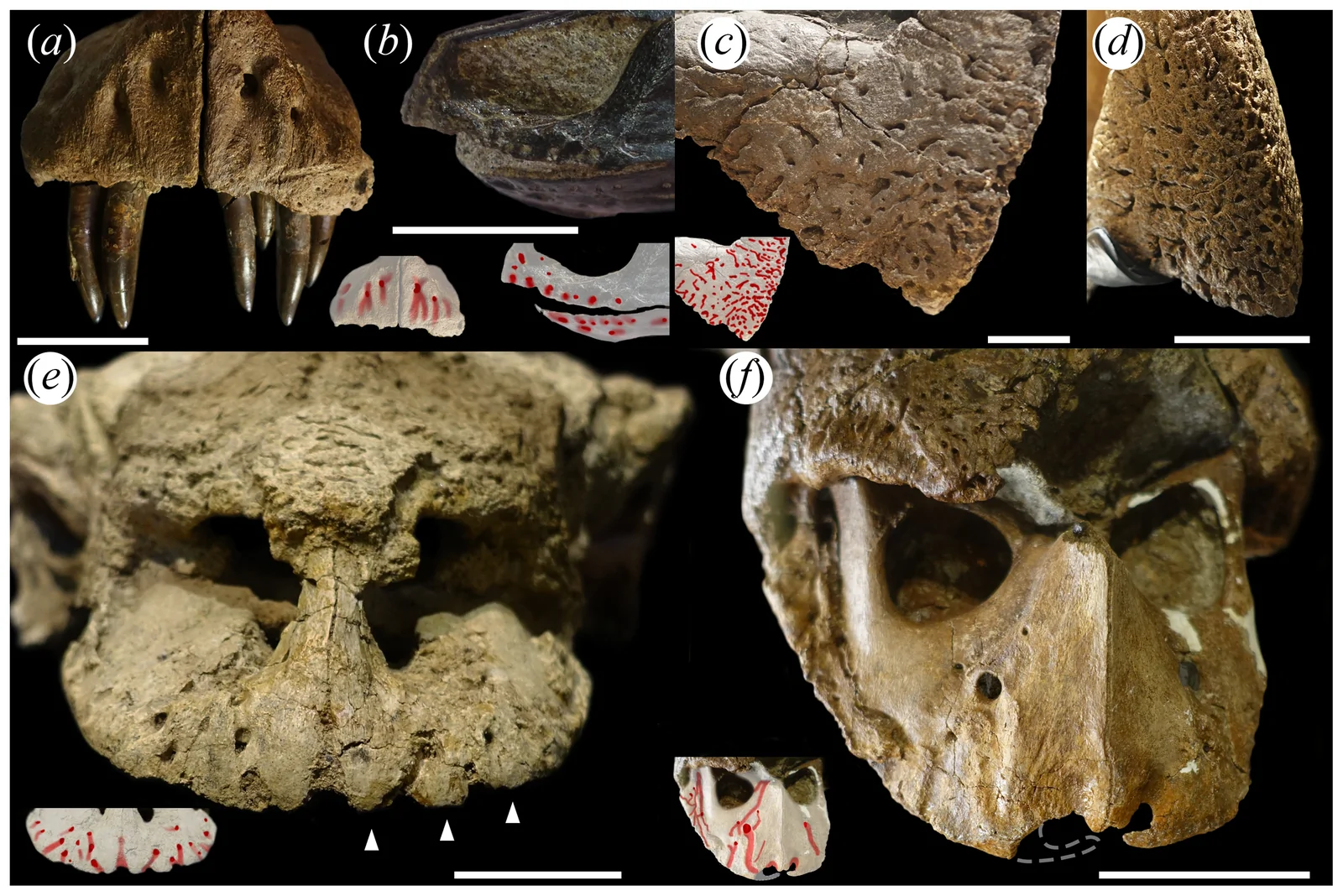
Comparison of the rostral region among Dinosauria, with miniatures highlighting foramina and associated neurovascular grooves. (*a*) Premaxillae of *Daspletosaurus torosus* TMP 2001.036.0001 in anterior view. (*b*) *Struthiomimus* sp. TMP 1990.026.0001 in dorsolateral view. (*c*) Rostral of *Triceratops horridus* TMP 2014.022.0022 in anterolateral view. (*d*) Rostral of *Centrosaurus apertus* TMP 1994.182.0001 in anterolateral view. (*e*) *Tsagantegia longicranialis* MPC-D 700/17, anterior view—notice the indentations along the tomial margin, creating denticle-like triangular projections highlighted with white arrows. (*f*) *Edmontonia rugosidens* TMP 1998.098.0001. Scale bars = 50 mm (*a, c–f*), 20 mm (*b*).

**Figure 5.**
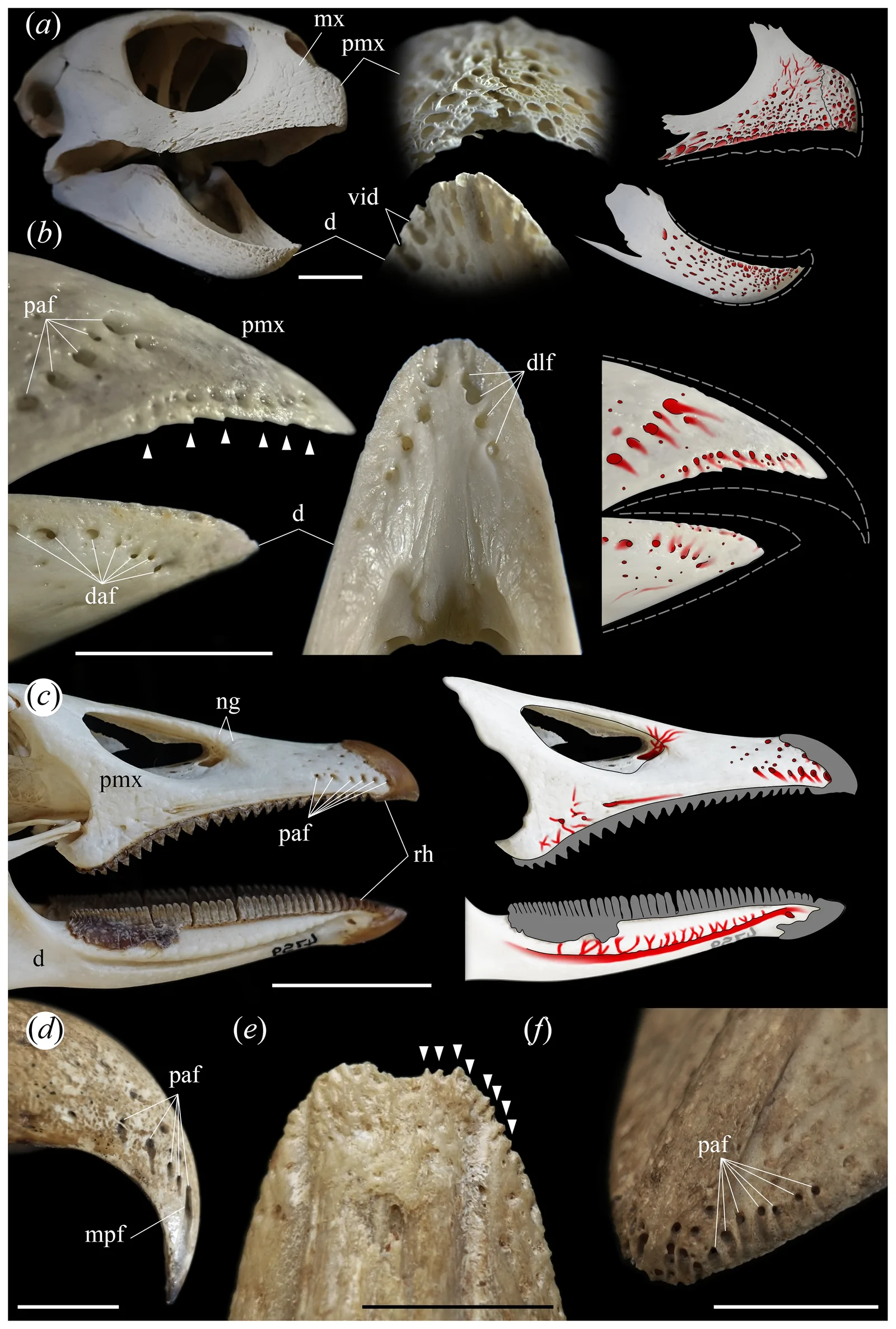
Osteology of the beaks in turtles and birds and diversity of the neurovascular structures. (*a*) *Caretta caretta*, with details of the bone porosity on the tip of premaxillae and dentary (centre), and schematic drawing of neurovascular foramina and associated grooves (right). Dotted lines represent the extent and shape of the rhamphotheca. (*b*) *Gallus gallus*, detail of the lateral perforations of the premaxilla and dentary (left), occlusal foramina of the dentary (centre) and schematic drawing of neurovascular foramina and associated grooves (right). Dotted lines represent the extent and shape of the rhamphotheca. (*c*) *Chen caerulescens* with preserved rhamphotheca, detail of the lateral neurovascular perforations of the premaxilla and dentary (left) and schematic drawing of neurovascular foramina and associated grooves (right). (*d*) *Diomedea* sp., hooked anterior extremity of the premaxilla. (*e*) *Rhea americana*, dorsal view of the anterior premaxilla sporting an irregularly denticulated margin. This specimen exhibits an anomalous morphology of the tomial margin, possibly linked to a traumatic event. (*f*) *Dromaius novaehollandiae*, anterolateral detail of the premaxilla. White triangles highlight denticle-like projections along the tomial margin. Abbreviations: d, dentary; daf, dentary terminal aligned foramina and associated grooves; dlf, dentary occlusal foramina and associated grooves; mpf, major terminal premaxillary foramen; mx, maxilla; ng, narial superficial grooves; paf, premaxillary terminal aligned foramina and associated grooves; pmx, premaxilla; rh, rhamphotheca; vid, vascular indentations. Scale bars = 20 mm (*a–c*), 10 mm (*d–f*).

**Figure 6.**
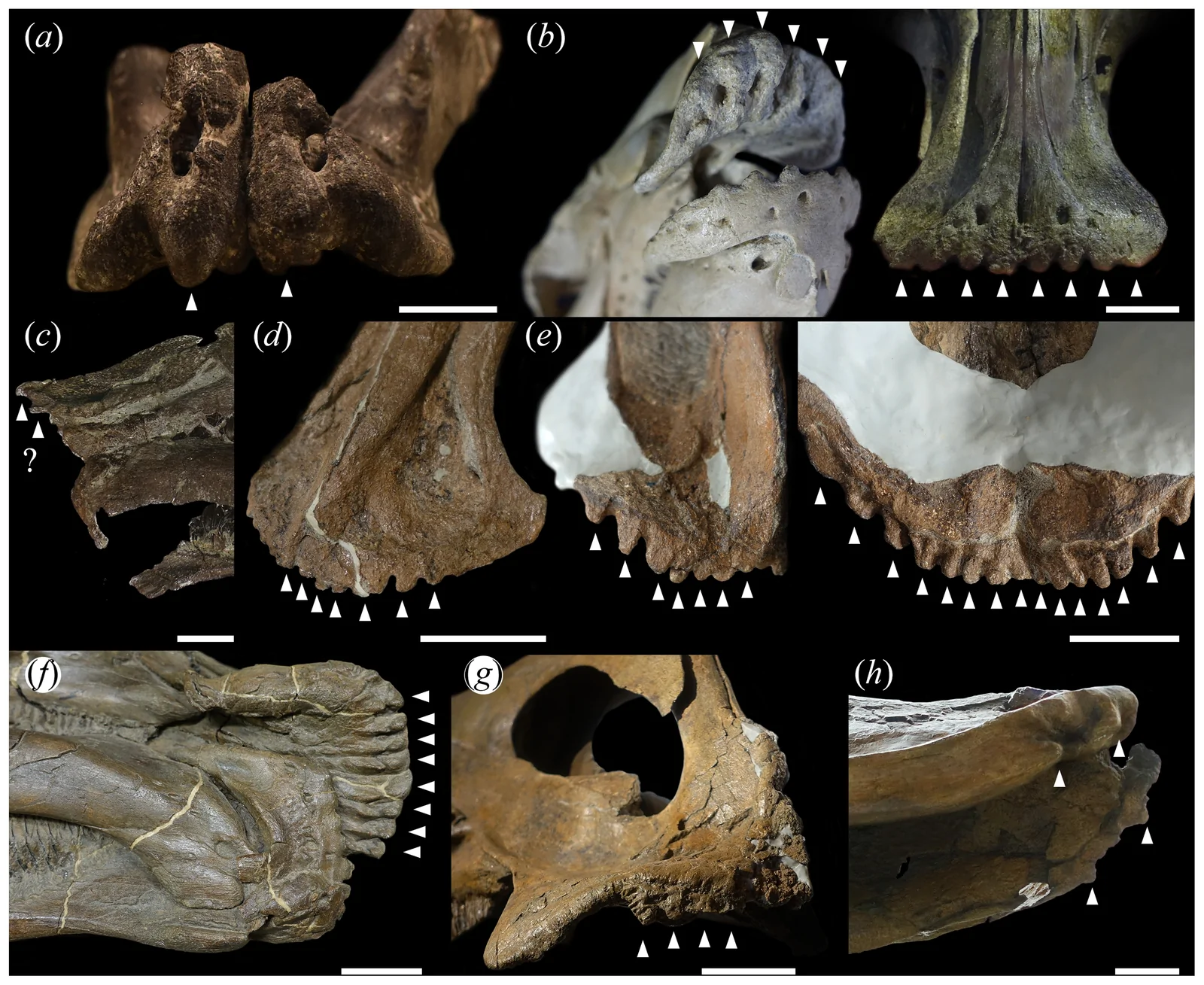
Details of premaxillary denticulations in ornithopods, highlighted by white triangles. (*a*) *Camptosaurus dispar* YPM 1880. (*b*) *Gobihadros mongoliensis* MPC-D 100/754 (left) and MPC-D 100/763 (right). (*c*) *Hypacrosaurus stebingeri* TMP 1989.079.0052, perinatal individual. (*d*) cf. *Hypacrosaurus* sp. UALVP 57103.01, juvenile individual. (*e*) *Lambeosaurus magnicristatus* UALVP 60000.00, anterior view of the right premaxilla (left) and ventral view of the articulated premaxillae (right). (*f*) *Prosaurolophus maximus* TMP 1998.050.0001. (*g*) *Brachylophosaurus canadensis* TMP 1990.104.0001. (*h*) *Saurolophus angustirostris* MPC-D 100/706. Scale bars = 10 mm (*a, c*), 20 mm (*b*), 50 mm (*d–h*).

#### 3.2.2. Preserved rhamphothecae in Ornithischia

Preserved rhinothecae are known in several hadrosaurid taxa, including *Edmontosaurus* (SMF R 4036, LACM 23502), *Brachylophosaurus* (JRF 115 [48]), *Maiasaura* (ROM 44770), *Parasaurolophus* (RAM 14000 [49]) and *Hypacrosaurus* (TMP 2013.025.0001 [50]). A preserved rhamphotheca was also described in *Corythosaurus* (CMN 8676 [51]) but has since been lost [12]. Notably, among other ornithischians, the rhinotheca is also preserved in the nodosaurid *Borealopelta* (TMP 2011.033.0001 [52]). All these taxa share a consistent morphology: large, dorsoventrally expanded beaks forming a marked overbite and often bearing vertical grooving (figure 7), representing either moulds of the internal surface or the external ornamentation (or both, depending on each case [12,50]). Unlike those of extant birds and chelonians and of exceptionally preserved non-avian theropods, these structures do not closely mirror the marginal morphology of the premaxillae. The premaxillary tomial edges are comparatively horizontal or distinctly anteriorly oriented, and grooves on the rhinothecae do not correspond to marginal denticulations.

**Figure 7.**
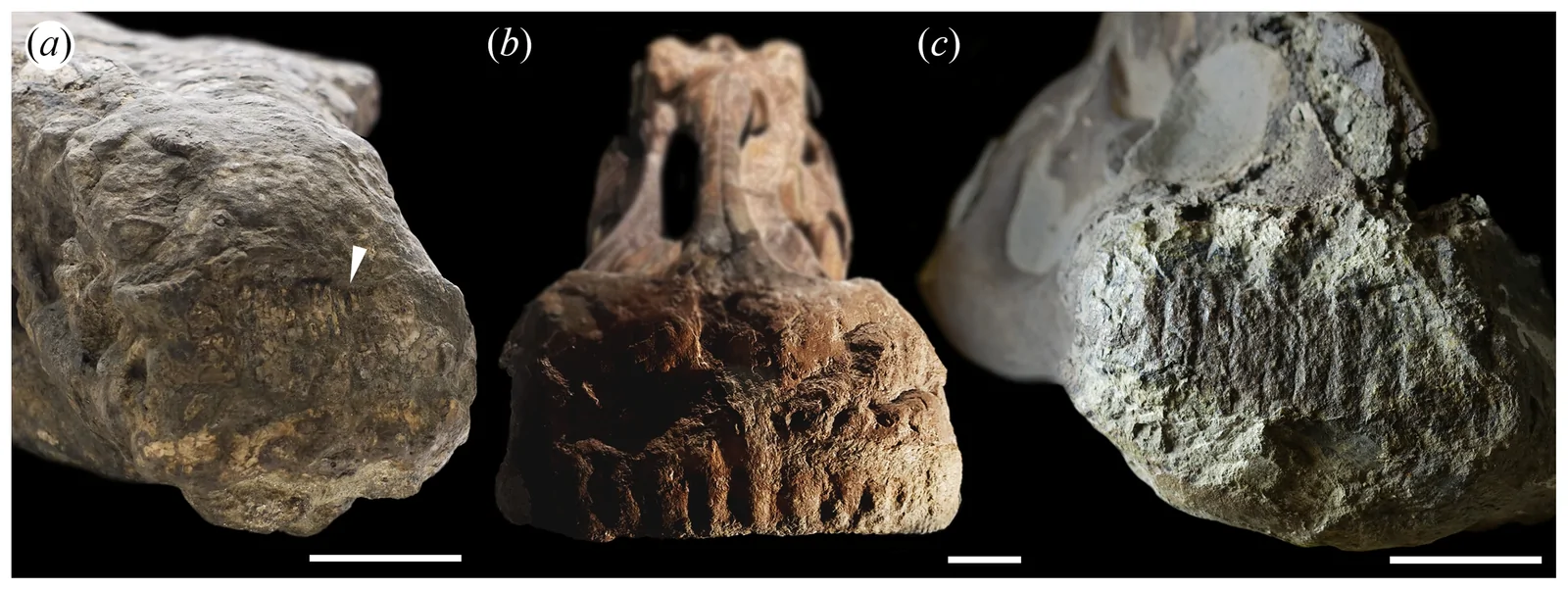
Detail of preserved rhamphothecae in an ankylosaur, a saurolophine and a lambeosaurine. (*a*) Rhinotheca of *Borealopelta markmitchelli* TMP 2011.033.0001. Notice the fine vertical grooving exposed in part of the beak, highlighted with a white arrow. (*b*) Rhinotheca of *Edmontosaurus annectens* SMF R 4036, courtesy of Riccardo Rocchi. (*c*) Rhinotheca of *Hypacrosaurus altispinus* TMP 2013.025.0001. Scale bars = 50 mm.

Hadrosauriform gnathothecae remain unknown. Referring to the well-preserved *Edmontosaurus annectens* LACM 23502, Morris [12] states, ‘a complementary structure that would have been attached to the lower mandible was not found even though conditions for its preservation must have been similar to those for the upper bill mold’, suggesting a smaller size and/or significantly different morphology. In taxa such as *Probactrosaurus* (PIN 2232/1-2, PIN 2232/23-1) and *Bactrosaurus* (AMNH 6575, AMNH 6372), dorsally oriented predentary denticles occlude into slots on the premaxillae [53], effectively functioning as occlusal pits. Predentary denticles recall the occlusal morphologies of giant herbivorous tortoises (e.g. *Centrochelys*) and probably reflect the morphology of the gnathotheca: localized porosity even in *Tethyshadros* may indicate a less voluminous keratin covering than that of the rhinotheca, with the effective corneous covering limited to a thin occlusal area similar to trionychid rhamphothecae. The geometrical and less variable shape of predentary denticles may suggest systematic roles in functional morphology. Similar general configurations occur in basal iguanodontians and derived hadrosaurids, indicating a trend towards enlarged overbiting rhinothecae increasingly decoupled from underlying bone morphology and more specialized gnathothecae with complex occlusal geometries.

#### 3.2.2. Rostrum osteology and associated soft tissues in extant Archelosauria

Within the comparative sample of extant taxa, the tomial margin of chelonians typically lacks well-developed denticles. In *C. caretta*, the entire lateral surface of beak-bearing bones (premaxillae, maxillae and dentaries) is uniformly perforated by a high density of neurovascular foramina and associated shallow anastomosing grooves (figure 5*a*). A series of posteriorly oriented aligned foramina follows the tomial margin of the maxillae and dentaries, while the same area in the premaxillae bears more chaotic perforations. Only millimetre-scale indentations, caused by marginal foramina, are present on both the maxilla–premaxilla and the dentaries. This condition is common for most living chelonians. Trionychids, on the other hand, have a markedly different perioral epithelium, with a dorsoventrally reduced rhamphotheca and the presence of voluminous soft perioral tissues. The maxillae and dentaries have relatively smooth surfaces which are perforated by fewer and larger foramina than in other turtles. The bone porosity increases towards the tomial margin, where aligned foramina subtly interrupt the continuity of the bone surface. The macromorphology of the rhamphotheca in chelonians generally reflects the morphology of the underlying bone, occasionally with additional small marginal denticulations or dorsoventral millimetric grooving on occlusal surfaces. Giant herbivorous tortoises (*Aldabrachelys*, *Centrochelys and Chelonoidis*) possess rhamphothecae with tomiodonts and/or elaborate serrations and relatively complex occlusal morphologies easily observable in living specimens; however, there is no proper description or morphofunctional analysis of these structures and the underlying bone in the literature. The three-dimensional organization of the neurovascular component underlying the chelonian beak is not properly documented; the density and orientation of foramina suggest the presence of a capillary network branching from larger vessels and bridging over the tomial margin. Accordingly, histology of post-hatching emydids shows a dense dermis in continuity with the periosteum, which is interrupted by the numerous bone porosities containing nourishing blood vessels [54]. Living crocodiles and alligators do not possess edentulous portions of the oral margin but share with other archosaurs an increased number of foramina on premaxillae, maxillae and dentaries. These are randomly distributed—except for a row of aligned ‘labial’ foramina extending along the dentigerous margin, sometimes poorly distinguished from other foramina. This profuse porosity houses both a dense network of vascular canals [55] and the specialized nervous terminations of the integumentary sensory organs [56]. Notably, the perioral epithelium in extant members of Crocodylia consists of a highly cornified rigid structure, and the scale-like polygonal pattern exhibited in these areas is not homologous to true scales, arising instead through mechanical cracking and folding of a continuous surface during embryonic development [57,58]. Birds represent the only extant beaked archosaurs and constitute the phylogenetically closest analogue of beaked non-avian dinosaurs. However, they display a staggering interspecific and intraspecific diversity of forms, with the neurovascular patterns also varying between different areas of the same beak [59] (figure 5; supplementary material, figure S2). A simplified condition typical of the average bird consists of two rows of major aligned foramina on the lateral surface of the premaxillae, one close to the tomial margin and one positioned slightly more dorsally, both associated with grooves leading to the tomial margin (figure 5*b–f*; supplementary material, figure S2). On the ventral surface of the premaxillae, several foramina and associated grooves also project anterolaterally towards the tomial edge. A single row of major aligned foramina is instead usually present on both the anterior lateral and medial surfaces of each dentary. In addition to these, a bundle of relatively deep ‘narial’ vascular grooves is commonly observed extending from the anterior margin of the naris on the dorsolateral surface of the premaxilla (figure 5*c*; supplementary material, figure S2a,e). As in turtles, the general shape of avian rhamphothecae mirrors the shape of the underlying bone, but they can also bear small, complex structures such as lamellae, serrations and denticles without any underlying osteological correlate. Conversely, osteological correlates are known (albeit not systematically documented) for certain regionalizations of the beak: anseriforms with a premaxillary ‘nail’ have a greater concentration of foramina in the corresponding areas (figure 5*c*; supplementary material, figure S2), while seabirds and raptors with prominent terminal ‘hooks’ have convergent rows of progressively larger foramina, sometimes (e.g. *Diomedea* and *Procellaria*) culminating in a large, terminal foramen with associated groove reaching the tomial apex of the premaxillae (figure 5*d*; supplementary material, figure S2). Neurovascular bundles are known to pass through premaxillary and distal dentary foramina into the dermis beneath the rhamphotheca. These neurovascular porosities contain both nerves and large blood vessels that nourish the stratum germinativum beneath the rhamphotheca. The three-dimensional structure of the nervous component is documented in detail [60], paralleling numerous extensions of the dermis (papillae) which project rostrally into the rhamphotheca from the tomial margin of premaxillae and dentary [61] (figure 8). The neural branches tracking the papillae house mechanosensitive Herbst and Grandry corpuscles, which are visible on the occlusal surface of the rhamphotheca as marginal pits connected to epidermal canals [60,61]. Although detailed data on the vascular organization are limited, the presence of large vessels branching towards the rhamphotheca and extending along the tomial margin is documented both in the available literature [61,62] and in the physical preparation of specimens. The bony tomial edge is continuous or only subtly interrupted by shallow vascular grooves in most analysed taxa (figure 5; supplementary material, figure S2), but some specimens have unequivocal small vascular indentations (figure 5*e*; supplementary material, figure S2e), and a full-grown *Gallus* in our sample shows anteriorly projecting ossified denticles corresponding to the basal portions of papillae (figure 5*b*).

**Figure 8.**
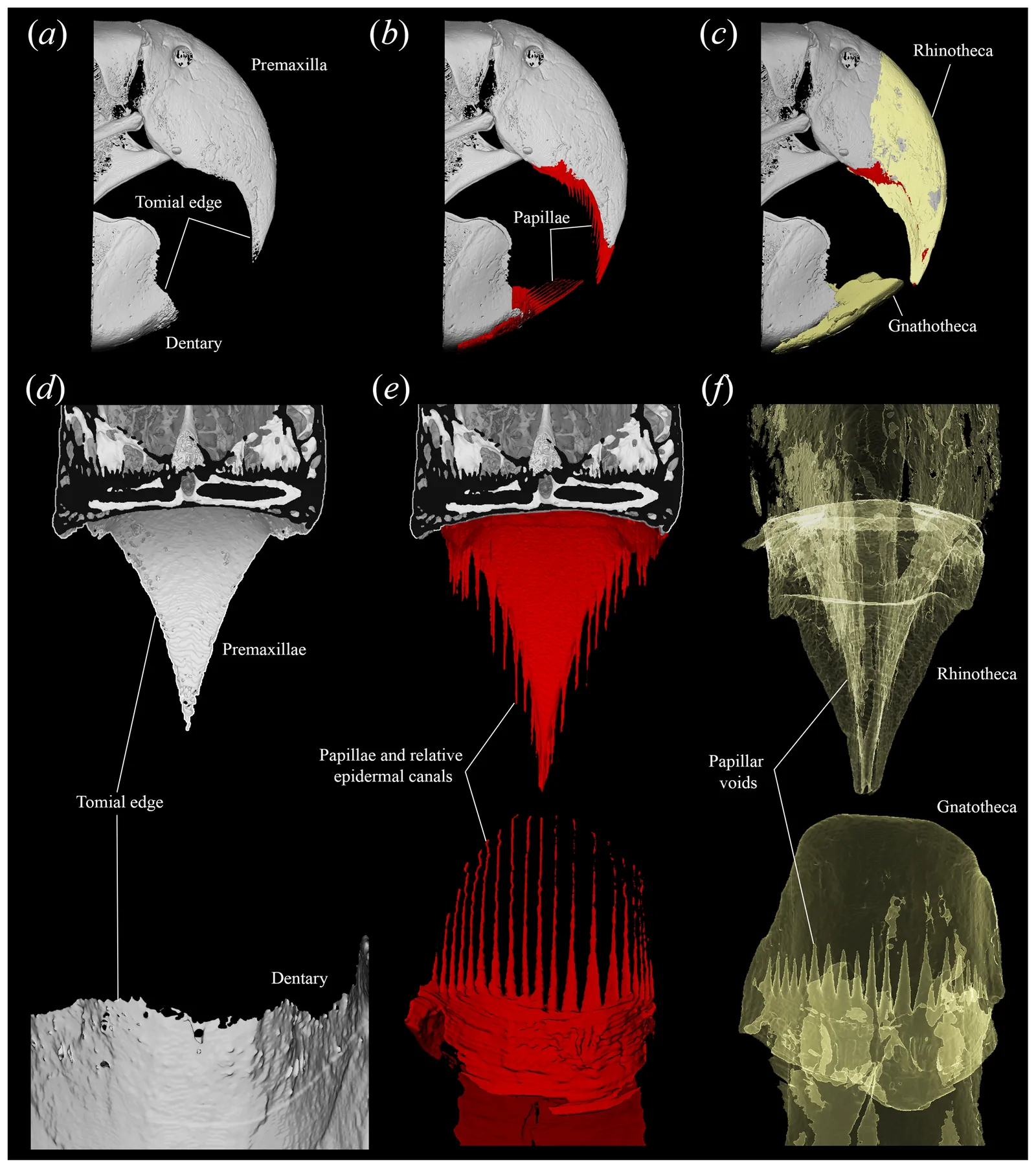
3D render of isolated papillae on *Ara macao* OUVC 10633. (*a–c*) Right lateral view of the rostral portion of the skull and mandible as they appear (*a*) without soft tissues, (*b*) with isolated papillae and (*c*) with overlying keratinous rhamphotheca. (*d–f*) Lingual view of the premaxillae and dentary (*a*) without soft tissues, (*e*) with isolated papillae and (*f*) with isolated rhinotheca and gnathotheca, exhibiting the inner voids left by papillae. Premaxillae and dentary in lingual views not to scale.

### 3.3. Rostral neurovascular structures in *T. insularis*

#### 3.3.1. Premaxillary neurovascular system

The left premaxilla of SC 57021 preserves superficial grooving that is continuous with the openings of the neurovascular canals identified in the CT data (figure 9*a–f*). Two shallow grooves on the laterodorsal and lateroventral surfaces of the bony naris, respectively, are interpreted as traces of the lateral nasal circulation. These vessels probably originated as the dorsal and ventral branches of the nasal circulation, travelling adjacent to the lateral surfaces of the premaxilla inside the bony naris: the dorsal and ventral branches probably anastomosed rostrally as in birds and other archosaurs [62–64]. The ventral branch reaches the ventrolateral margin of the naris and then exits the naris laterally. The dorsal branch bifurcates towards the rostral portion of the naris, with a more dorsal portion exiting the naris laterally, and a more ventral one entering the premaxillary canal. The inner section of the neurovascular voids of the premaxillae connects anterolaterally with all the major premaxillary foramina, medially to the medial sinus through a foramen and ventrally to the palate through a vertical foramen. Superficial grooves departing from the three major premaxillary foramina branch variably and lead to the indentations between the anterior premaxillary denticles (figure 9*a–f*); the groove from the medialmost foramen is directed medially and leads into the premaxillary suture. These morphologies thus define a series of circumflex vessels wrapping around the tomial edge of the premaxilla, each leading ventrally to the rostrocaudal grooves which are interpreted as correlates for a developed palatine circulation [62,64]. In dorsal and ventral view, the lateral margins of the medial and central denticles display subcircular indentations which probably represent the lumens of more anterior vessels belonging to the same complex (figure 2*f*). The circumflex vasculature probably extended up to the anterior margin of the premaxillary denticles, filling the empty indentations and nourishing a continuous stratum germinativum for the growth of the rhinotheca. The lateralmost groove leads to the vertical palatal foramen connecting the lateral branch of the palatine circulation to a supposed secondary branch of the ventral lateral narial circulation.

**Figure 9.**
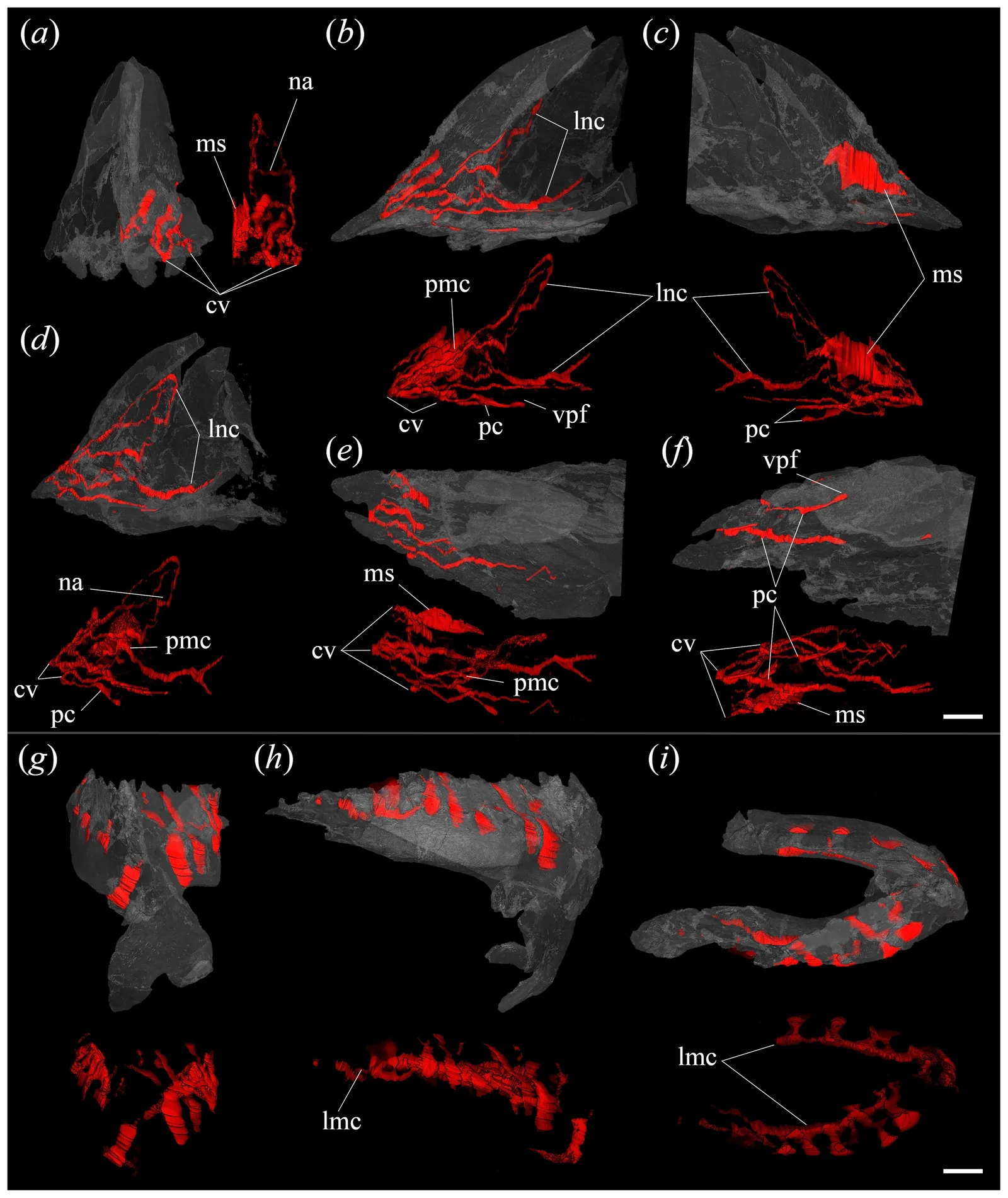
3D renders of the isolated premaxillary (*a–f*) and predentary (*g–i*) neurovascular network. (*a*) Premaxillary neurovascular structures in frontal view, with bones (left) and isolated (right). (*b*) Lateral left view, with bones (top) and isolated (bottom). (*c*) Lateral right view, with bones (top) and isolated (bottom). (*d*) Left caudolateral view for better assessment of the anterior narial correlates, with bones (top) and isolated (bottom). (*e*) Dorsal view, with bones (top) and isolated (bottom). (*f*) Ventral view, with bones (top) and isolated (bottom). (*g*) Predentary neurovascular structures in anterior view, with bone (top) and isolated (bottom). (*h*) Lateral right view, with bone (top) and isolated (bottom). (*i*) Dorsal view, with bone (top) and isolated (bottom). Abbreviations: cv, circumflex vessels; lmc, lateral mandibular circulation; lnc, lateral narial circulation; ms, medial sinus; na, narial anastomosis; pc, palatal circulation; pmc, premaxillary canal; vpf, vertical palatal foramen. Scale bars = 10 mm.

#### 3.3.2. Predentary neurovascular system

CT data show that the predentary bone tissue of SC 57021 is volumetrically dense and relatively homogeneous. It lacks significant inner cavities except for a possible small medial chamber, located just dorsal to the peduncle of the bilobate process. However, superficial grooving and mediolateral foramina are consistent with the identification of a pair of large ventral vessels, probably originating from the large ventrolateral foramen near the symphyseal process of each dentary [65], which extend laterally to the bilobate process towards the two larger, anterior foramina (figure 9*g–i*). These foramina allow the vessels to pass through the predentary to the medial surface, where they extend caudally into a large symmetric groove along the caudal processes. The configuration of this groove resembles the lateral mandibular vascularization of birds [63] and connects to the row of large mediolateral foramina. From these foramina, multiple branches of the larger vessel reach the labial surface, where they branch and anastomose variably both ventrally and dorsally. Dorsally, the subtriangular denticles of the predentary exhibit a progressively higher porosity and density of smaller foramina towards their apices, suggesting a more direct role in supporting the gnathotheca.

## 4. Discussion

### 4.1. Vascularization

The rostral neurovasculature of *Tethyshadros* is similar to that of extant chelonians and birds and is consistent with available literature [62,64] regarding this anatomical region in some dinosaur clades (Sauropoda, Ankylosauria, Pachycephalosauria, non-avian theropods, Aves [64]), crocodilians (*A. mississippiensis* [55]) and even squamates (*Iguana iguana* [66,67]). Following the actualistic model provided by living archosaurs and squamates, the same vascular configuration—especially in the conservative narial region—has recently also been supported for ceratopsians (*Triceratops* [68]) and the Triassic pseudosuchian *Riojasuchus* [69]. This is the first detailed description of these structures in ornithopods. In well-preserved specimens of other taxa, such as *Gobihadros* and *Choyrodon* [36], surface grooves inside the bony naris follow the same pattern observed in *Tethyshadros*. Later-diverging hadrosaurs have less visible correlates, probably influenced by the broader morphology of the premaxillae and well-developed labrum, but the consistent position of the premaxillary canal suggests the same configuration. The putative nasal neurovasculature of ceratopsians also provides a strikingly similar model, with nasal circulation having an enhanced role compared to the maxillary one [68]. Assuming that the anterior maxillary foramen in hadrosauroids housed large rostrally directed maxillary vessels in addition to the maxillary nerve, these would have encountered a continuous surface and lateral protrusion at the contact with the premaxilla and thus would have needed to arch below the oral margin and reach the rostralmost region either by entering the naris laterally or ventrally, suggesting that the palatine circulation was better suited to providing the main vascular supply to the premaxillae. Crocodilians (e.g. *Alligator* [55]) have a very similar narial circulation, but birds offer the closest analogue among extant taxa [62,63], displaying a notably similar architecture of the lateral narial circulation and rhamphotheca feeding vessels. In birds, the palatine circulation has an enhanced role in both thermal exchange and blood supply for the rhamphotheca, with the palatine artery entering the premaxilla anteriorly, and anastomosing with both branches of the lateral nasal artery. The venous plexus of the palate–nasal complex follows a parallel course, with anastomosis between palatal and nasal veins [62]. The palatal and narial regions ultimately function as a key contact area for the respective circulations, allowing for both increased blood supply to the premaxillae and enhanced thermal exchange efficiency by draining cooled blood towards the brain. The inferred neurovascular pathways in *Tethyshadros* strongly suggest the same set of adaptations.

### 4.2. Functional morphology of the premaxillary denticles

The form and distribution of denticles, foramina and associated grooves in *Tethyshadros* suggest that the premaxillary indentations arose through two non-exclusive processes: (i) excavation by enlarged circumflex vessels coursing within deep grooves linked to dorsally positioned foramina and incising the oral margin of the premaxilla; and (ii) dermal ossifications associated with papillary structures underneath the beak.

The superficial grooves are osteological correlates of a major neurovascular system, as discussed above and reported in the literature [64]. Their continuity with large foramina and their placement beneath the inferred rhamphotheca indicate a highly vascularized dermis supplying the developing cornified sheath. While the organization of these structures matches that observed in living chelonians and birds, their relative volume—relative to the inferred size of the corneous beak—is unparalleled among extant beaked reptiles. Accordingly, in hadrosaurids, the macromorphology of every known preserved rhamphotheca differs substantially from that of the corresponding premaxilla. This suggests that despite sharing a basic structural organization with birds, the derived anatomy of the hadrosauroid beak has no analogue among oral epithelia of extant species. In addition, bony denticulation may represent ossified papillary dermal structures analogous to those known in living birds. In extant birds, the papillae can display triangular morphologies recalling the ossified projections observed in ornithischians (*Psittacus erithacus* [60]; *A. macao*, figure 8; Galloanserae, supplementary material, figure S3). Given their dermal origin, ossification of such papillae is developmentally plausible as an extension of the dermatocranial premaxilla, and one *Gallus* specimen in our comparative sample appears to show an incipient form of this exact phenomenon (figure 5*b*). Evidence from crocodilian osteoderms suggests that postnatal dermal ossification begins with a highly vascularized dermis and transitions through fibrotic to cellular phases via intramembranous ossification [70]. Available data indicate that *Tethyshadros* and other hadrosauroids may have followed similar ontogenetic trajectories concerning premaxillary denticles: perinatal individuals lack denticulation, juveniles and subadults exhibit the most pronounced structures, and adults show proportionally thicker, more closely spaced projections [20,40–45]. This pattern may suggest a postnatal ossification comparable to that of crocodilian osteoderms and could have functional implications, with premaxillary denticulation potentially informative for assessing ontogenetic stage in ornithopods. Ongoing research involving denticle histology should clarify their developmental origin (G. Sukkert 2026, personal communication).

A pronounced somato-sensory role for the hadrosauroid beak would be consistent with the mechanosensory function of papillae in avian beaks [71–74], which was probably inherited from non-avian theropod ancestors [72]. The origin of beaks in theropods and ornithischians was a product of evolutionary convergence; however, a shared, highly developed sensory capacity of the beak shared by theropods and hadrosaurids may suggest that the anatomical substrate enabling such specialization was widespread across Dinosauria prior to the origin of beaks. As beaks evolved independently multiple times among dinosaurian clades [9,10], somato-sensory predispositions may have promoted repeated convergence on sensitive, versatile cornified rostra.

Both hypotheses—vascular excavation and dermal ossification—converge on a key implication: the tomial edge of the premaxilla in *Tethyshadros* does not faithfully reflect the external morphology of the rhinotheca. The large neurovascular foramina, extensive bone porosity, reconstructed vascular networks and inferred possible papillary development collectively support the presence of a substantially expanded, highly developed beak, comparable in complexity to that of derived hadrosaurids. Interestingly, similar combinations of osteological correlates and morphologies can be observed in other regions of the amniote skeleton supporting large keratinous structures, representing the only other available analogous models. The best-documented potential functional analogue is found in the vascular organization of ungual phalanges, both in large ornithischians and in extant large mammals bearing hooves or hoof-like structures (figure 10). In large, extensively studied domestic ungulate mammals such as *Equus* and *Bos* (figure 10*a–c*), complex vascular networks branch both internally and superficially across the keratin-bearing regions of ungual phalanges. Remarkably, a series of circumflex vessels arches dorsoventrally around the ungual margins [75] in a configuration comparable to the marginal beak vasculature of birds and *Tethyshadros*. The arching vessels concentrate in serial bundles that pass through discrete concavities along the bone margin, which is scalloped with variably prominent ‘denticles’. For example, proboscidean unguals exhibit marginal denticulation-like features that closely resemble premaxillary denticles in some hadrosaurian beaks (figure 10*d*). Such osteological features are replicated on ornithischian hoof-like ungual phalanges, suggesting a comparable vascular distribution. This assumption is supported by preserved keratinous sheaths in *E. annectens* which demonstrate that the cornified covering was remarkably hoof-like and extended well beyond the underlying bone [76]. Because hooves and nails in large terrestrial amniotes undergo continuous wear, their keratinous coverings greatly exceed the dimensions of the supporting phalanges, whose finer morphology remains concealed beneath soft tissues. Consequently, external corneous structures only broadly reflect the underlying bone and may differ substantially in form, as seen in proboscideans (figure 10*d*). These observations might suggest that very similar functional configurations are selectively favoured in the evolution of proportionally large, evergrowing, rapidly worn keratinous structures.

**Figure 10.**
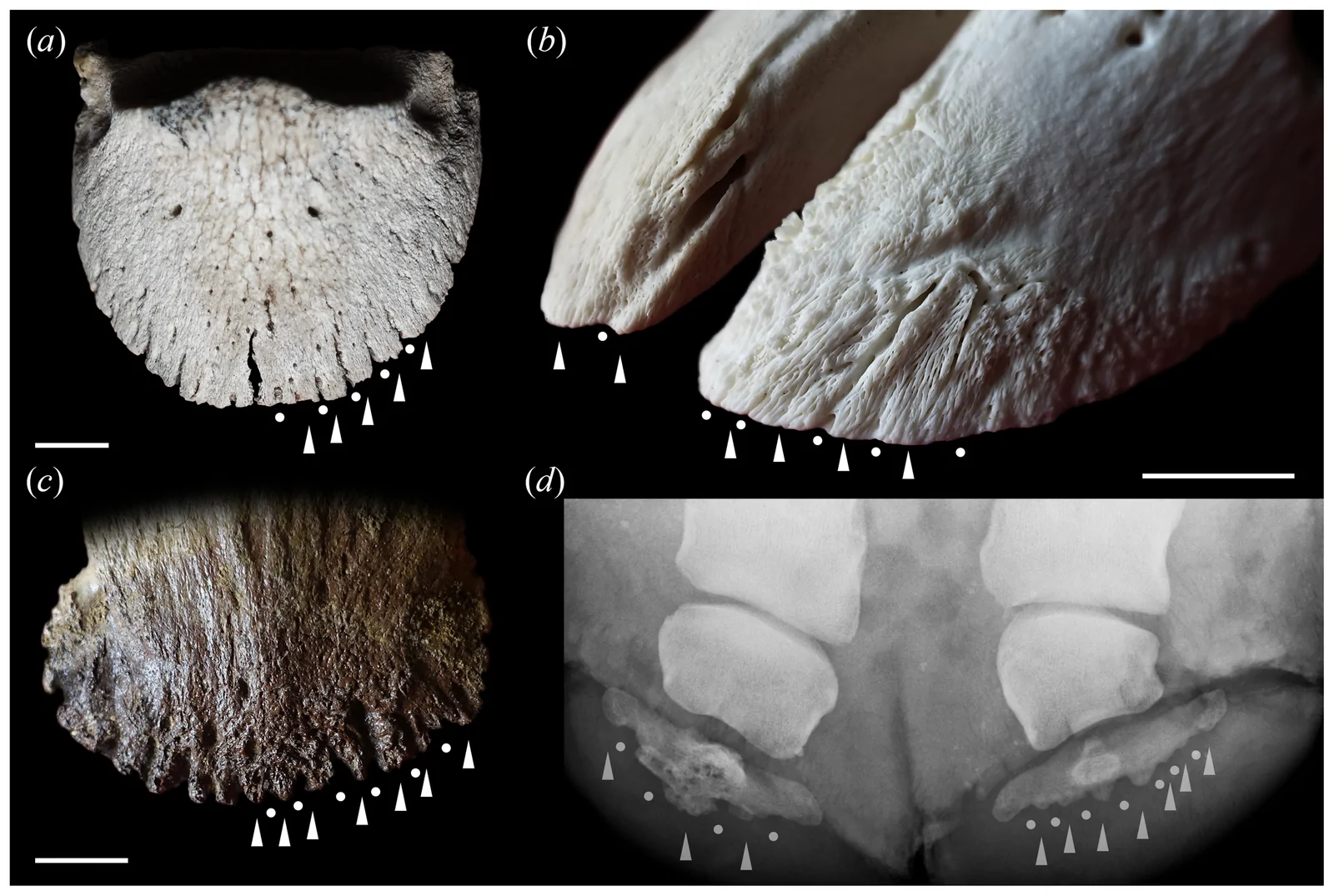
Comparable denticle-indentation morphologies across ungual phalanges. (*a*) *Equus ferus*. (*b*) *Bos taurus*. (*c*) Ornithischia indet. (*d*) *Elephas maximus* (with permission, picture taken by Dr J. R. Hutchinson). Vascular indentations and relative projections/denticles are highlighted with white dots and white triangles, respectively. Scale bars = 10 mm.

### 4.3. Functional implications for food processing

An enlarged and morphologically elaborate rhamphotheca may have supported several functions (including socio-sexual display [77]), but food acquisition and processing are the most readily inferred. Rhamphothecae play key roles in food acquisition in birds and chelonians, driving dietary shifts during ontogeny and underlying diversification in some groups [78–82]. Ornithischians and beaked non-avian theropods probably underwent comparable dynamics. Although hadrosauroids evolved highly efficient dental batteries with continuous tooth replacement [21,83–85], the beak's contribution to feeding ecology has been comparatively overlooked, partly because, among megaherbivores, no modern analogue combines a massive keratinous beak with complex dentition specialized for oral food processing.

An intrapremaxillary depression, probably housing a vascular sinus or neurosensory ganglion, represents an additional overlooked functional component that is widespread among archosaurs. Such proprioceptive structures are documented at the mandibular symphysis in balaenopterid whales [86] and in *A. mississippiensis* [87], where they detect mechanical stress and torsional forces. In *Tethyshadros*, such a system could have acted as an effective detector of mechanical stress, particularly given the specialized masticatory adaptations and proposed cranial kinesis of hadrosauroids. Together with tactile papillae, the hadrosauroid beak could therefore have functioned as a highly proprioceptive organ, functionally analogous to that of birds.

Osteological correlates in *T. insularis* indicate a large, robust rhamphotheca suited to cropping coriaceous vegetation and/or tolerating high abrasion rates. The occlusal surface on the mandibular tooth battery of specimen SC 57026 exhibits deep mesowear striae oriented mainly craniocaudally, with a secondary oblique component (figure 11). This bimodal orientation indicates primarily palinal jaw movements, with a secondary orthal component, consistent with the various biomechanical models proposed for hadrosaur mastication dynamics [84,85,88–90]. Striae exceeding 150 µm in width suggest processing of extremely abrasive material. Such wear probably reflects ingestion of coarse exogenous grit particles on the order of approximately 100–200 µm [91] rather than phytoliths alone. In the coastal environment characterized by carbonate bedrock and alluvial calcrete inhabited by *Tethyshadros*, limestone particles within soils could have been abundant, particularly under strong seasonality. Elevated environmental grit loads are known to intensify dental abrasion in herbivores from arid or karstic settings [92–94].

**Figure 11.**
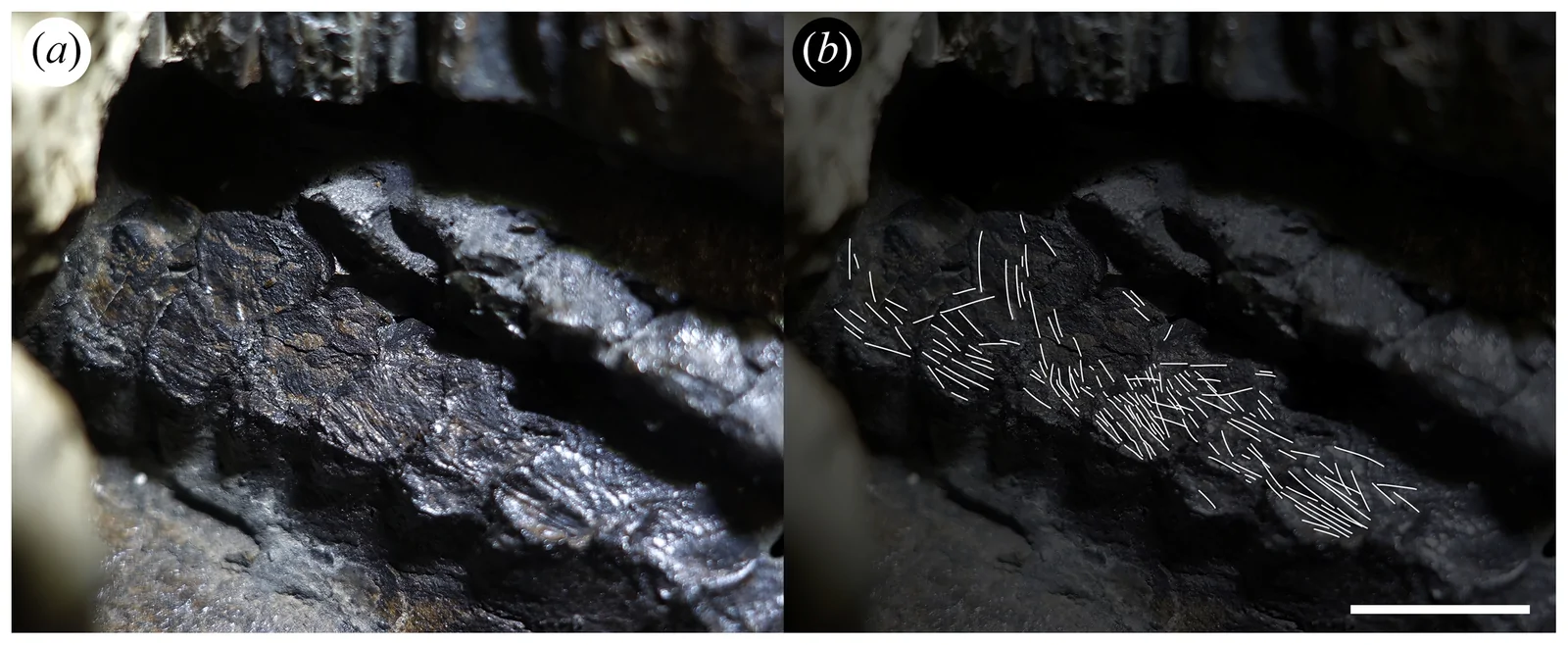
(*a*) Detail photo of the mesowear on the occlusal surface of the left dentary teeth in SC 57026. (*b*) Highlighted main scratches. Scale bar = 10 mm.

Within this system, dental batteries processed plant matter and sediment, while the rhamphotheca served as the primary cropping interface. Given the relative softness of keratin, intense wear would have required compensatory enlargement and rapid growth, functionally comparable to evergrowing rodent and lagomorph incisors or ungulate hooves [95,96].

### 4.4. Thermoregulation

Osteological correlates of cranial vasculature have received renewed attention for their thermophysiological implications, particularly in the oro-narial region [62,64–66,68,97,98]. In extant birds, the rhamphotheca can function as an efficient thermal radiator: the highly vascularized bill of *Ramphastos toco* is capable of dissipating substantial body heat [97]. More broadly, bill and narial morphology in birds reflects not only feeding ecology but also climatic adaptation [62,99,100]. Across diapsids, principal sites of cranial heat exchange include the nasal, oral and orbital regions, and blood flow can be preferentially directed to these areas to regulate temperature and protect the central nervous system from hyperthermia. Two strategies have been proposed: distributed thermoregulation, with more uniform vascularization across exchange regions, and focused thermoregulation, characterized by intensified blood flow in specific sites [64]. *Tethyshadros* lived in a warm, low-latitude environment [101,102] with documented tropical/monsoonal regimes [103]; given its comparatively large size [14] and inferred endothermic homoeothermy [104,105], it probably relied on focused thermoregulation. Its pronounced rostral vascularization supports a directional blood-flow pattern centred on the high blood supply demanded by the rhamphotheca and its close connection with narial circulation.

A proportionally large, densely vascularized and fast-growing rhamphotheca may therefore have supported not only feeding and social behaviour but also heat-dissipation strategies based on narial and oral ventilation, particularly in taxa lacking alternative radiators such as osteoderms, sails or cranial crests [106]. Narial ventilation has been proposed as a significant cranial heat exchanger in dinosaurs, and the enlarged nasal regions of hadrosaurs have long been implicated in thermoregulation alongside display and acoustic functions [107]. *Tethyshadros* and other early-diverging hadrosauroids lacked elaborate crests or hypertrophied narial surfaces but retained enlarged, teardrop-shaped nares. Expanded narial regions also evolved independently in basal iguanodontians reaching gigantism [108,109], reinforcing a link between nasal expansion and body mass.

Vascular grooves associated with the lateral narial circulation are preserved in several hadrosauroids such as *Gobihadros*, with deep narial grooves converging on the premaxillary foramen, which exchanged blood flow with the bill and anterior palatine region, mirroring the pattern inferred for *Tethyshadros* (figure 12*a,b*). In this model, arterial blood reached the premaxilla from both palatine and narial circulation to nourish the growing rhinotheca; venous return, partially cooled within the beak, then passed through the ventilated narial region for additional heat exchange before draining towards the neurocranium (figure 12). Such a pathway would have reduced endocranial temperatures directly or indirectly, enhancing thermal protection of the brain, a model shared to various degrees with other dinosaurs and reptiles [63,65,68].

**Figure 12.**
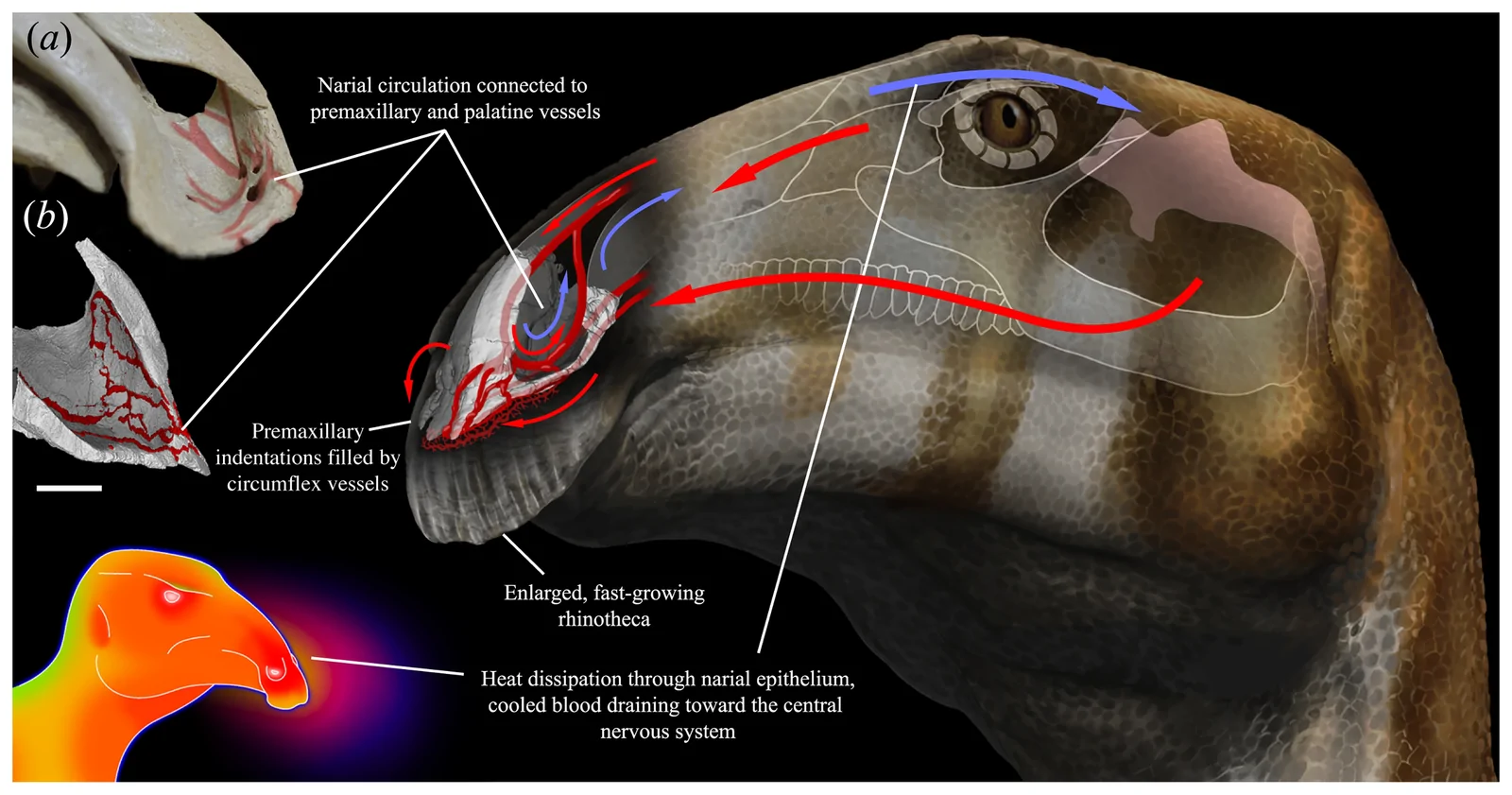
Schematic rendition of the inferred beak–nares thermal exchange system in *Tethyshadros*, with arterial/warm blood (red) nourishing the hypertrophied rhinotheca and passing through the naris, where venous/cooled return (blue) drains towards the neurocranium. (*a–b*) Highlight of osteological correlates for the narial premaxillary circulation in (*a*) *Gobihadros* and (*b*) *Tethyshadros*. *In vivo Tethyshadros* rendition by Davide Bonadonna. Scale bar = 20 mm.

## 5. Conclusions

The spike-like marginal denticulations and associated porosity of the premaxillae and predentary of *T. insularis* are here interpreted as osteological correlates of an exceptionally developed neurovascular network, rather than anomalous ‘spiked’ beak ornamentation. As this condition is well documented across hadrosauroids and earlier diverging iguanodontians, the rostrum of *Tethyshadros* emerges not as an osteologically aberrant feeding tool, but as the expression of a deeply integrated soft-tissue system whose evolutionary roots predate the rise of hadrosaurid gigantism [14].

Reconstruction of the vascular architecture reveals an extensive, integrated circulation linking the lateral narial region, palate, rhamphothecal substrate and a previously unrecognized median premaxillary sinus, with large circumflex vessels tracking the tomial margin. These vessels probably underpinned the marginal denticulations and supported a hypertrophied, rapidly renewing keratinous rhinotheca. Comparisons with extant systems sustaining evergrowing, high-wear keratinous structures reinforce this interpretation, as does the growing fossil record of preserved hadrosaur rhamphothecae, whose size and morphology routinely exceed and depart from the contours of the underlying bone. The coarse mesowear textures of the mandibular batteries in *Tethyshadros* further attest to the mechanical demands placed upon this beak during the processing of grit-laden, abrasive vegetation within karstic environments.

Beyond mechanical function, the denticulate rostral margin may have housed dermal papillae analogous to those of avian beaks, implying a tactile and proprioceptive capability rarely considered in ornithopods. Ontogenetic variation in hadrosauroids supports postnatal ossification and suggests that the beak functioned as a sensory interface during food selection as much as a cropping organ, potentially capable of fine environmental discrimination during foraging.

Critically, the scale of the rostral circulation in *Tethyshadros* has thermophysiological implications. Continuity between premaxillary and narial blood flow supports the existence of a unified heat-exchange system in which arterial supply to the rhinotheca was followed by staged venous cooling across the beak and nasal passages before reaching the braincase. In a large-bodied herbivore inhabiting tropical climates, such a mechanism would have mitigated cranial hyperthermia. We propose that this ancestral beak–nares heat-exchange module later diversified and enabled hadrosaurids to surpass a critical body-mass threshold: lambeosaurines elaborated the nasal circuit into crest-borne passageways, whereas saurolophines expanded the narial region to accommodate enlarged vascular soft tissues.

Taken together, these findings support a re-evaluation of the hadrosauroid beak as a large, dynamic, vascularized, sensory and thermoregulatory organ, functionally and evolutionarily comparable in complexity to the avian bill. The emergence of this multifunctional beak–nares complex may represent a key innovation that facilitated dietary versatility, environmental responsiveness and ultimately the ecological expansion and body-size escalation that characterize later diverging hadrosaurids.

## Supplementary Material



## Data Availability

Full CT scans and derived models discussed in the article are uploaded on MorphoSource, parent media ID 000821984 (premaxillae) and 000822290 (predentary) as reported in the ‘Material and methods’. These data will be visible on MorphoSource with restricted download. Supplementary material is available online [110].
